# Impact of surgeon caseload, case mix, and team composition on gender disparities in cholecystectomy outcomes

**DOI:** 10.1093/bjsopen/zraf179

**Published:** 2026-03-27

**Authors:** My Blohm, Riccardo LoMartire, Gabriel Sandblom, Lars Enochsson, Johanna Österberg

**Affiliations:** Department of Clinical Science and Education, South General Hospital, Karolinska Institutet, Stockholm, Sweden; Department of Surgery, Mora Hospital, Mora, Sweden; Centre for Clinical Research Dalarna, Uppsala University, Falun, Sweden; Centre for Clinical Research Dalarna, Uppsala University, Falun, Sweden; School of Health and Welfare, Dalarna University, Falun, Sweden; Department of Clinical Science and Education, South General Hospital, Karolinska Institutet, Stockholm, Sweden; Department of Diagnostics and Intervention, Surgery, Umeå University, Umeå, Sweden; Department of Clinical Science, Intervention and Technology, Division of Orthopaedics and Biotechnology, Karolinska Institutet, Stockholm, Sweden; Department of Clinical Science and Education, South General Hospital, Karolinska Institutet, Stockholm, Sweden; Department of Surgery, Mora Hospital, Mora, Sweden; Centre for Clinical Research Dalarna, Uppsala University, Falun, Sweden

## Abstract

**Background:**

Previous research has suggested that female surgeons achieve better outcomes in various procedures, but the underlying reasons remain unclear. This study aimed to explore potential explanations for observed gender disparities in cholecystectomy outcomes.

**Methods:**

A follow-up analysis was conducted using a previously reported population-based cohort from the Swedish Registry of Gallstone Surgery. All patients who underwent cholecystectomy in Sweden between 2007 and 2019 were included, with a subgroup (2017–2019) analysed for case mix. The association between surgeon gender and outcomes was assessed, considering caseload, case mix, team composition, and surgeon–patient gender dynamics.

**Results:**

The analysis included 150 509 cholecystectomies performed by 2555 surgeons (849 women, 1706 men). Female surgeons had fewer complications across all experience categories. The gender disparity in bile duct injuries was most notable among male surgeons with < 3 years of experience (risk ratio 1.77, 95% confidence interval 1.30 to 2.40). After adjusting for case mix, male surgeons had higher risks of surgical complications (risk ratio 1.28, 95% confidence interval 1.12 to 1.45), bile duct injuries (risk ratio 1.63, 95% confidence interval 1.01 to 2.62), and total complications (risk ratio 1.10, 95% confidence interval 1.03 to 1.19), but shorter operating times. Teams with only male surgeons had the highest complication rates. Women experienced the best outcomes when treated by female surgeons.

**Conclusion:**

Female surgeons were associated with more favourable cholecystectomy outcomes. These differences persisted after accounting for caseload, case mix, and team composition. Inexperienced male surgeons had a higher risk of bile duct injury, underscoring the importance of rigorous mentorship during the early years of practice.

## Teaser

This follow-up study examined gender disparities in cholecystectomy outcomes in a Swedish cohort, accounting for surgeon caseload, case mix, and team composition. Female surgeons achieved better outcomes even after adjustment, whereas low-volume male surgeons and all-male teams showed higher risks of bile duct injury.

## Introduction

Growing evidence over the past decade has shown that patients treated by female surgeons may have improved clinical outcomes. Several large-scale studies^1–7^ have demonstrated that procedures performed by female surgeons are associated with lower rates of short- and long-term complications, lower mortality rates, and fewer readmissions. However, the underlying reasons for these differences remain elusive.

Building on this existing body of evidence, a comprehensive analysis of patient outcomes following cholecystectomy procedures performed by female and male surgeons in Sweden has been conducted^1^. The previous study, published in 2023, demonstrated that female surgeons achieved significantly better outcomes regarding surgical complications, bile duct injuries, total complications, conversion rates, and length of hospital stay, compared with their male counterparts, despite slightly longer operating times. However, that analysis did not address the underlying reasons. The results aligned with those of Wallis *et al*.^7^, whose work was published simultaneously and reported fewer postoperative adverse outcomes at 90-day and 1-year follow-ups.

Despite consistency with previous research, the presence of gender-related differences remains contentious; some have argued that differences in caseload may contribute to these findings, whereas others have suggested that male surgeons manage more intricate procedures^8^. Other factors, such as team composition and patient–surgeon relationships, may influence outcomes^9–11^. Additionally, the obstacles women face in pursuing a surgical career may lead to a more selective group of female surgeons, emphasizing the need to expand female surgical resident recruitment^6^. However, most of these arguments are based on anecdotal evidence.

This study aimed to further analyse the previously published cohort^1^ to explore potential explanations for observed gender disparities in cholecystectomy outcomes by surgeon gender in Sweden. It was hypothesized that variations in surgical caseload play a key role in these disparities. Additionally, other commonly proposed factors such as case mix and team composition were investigated to provide evidence-based insights into these differences.

## Methods

### Study design

This register-based cohort study encompassed all operations recorded in the Swedish Gallstone Surgery and Endoscopic Retrograde Cholangiopancreatography Registry (GallRiks) from 2007 to 2019. The study was a follow-up analysis building on earlier findings to examine factors influencing gender disparities in cholecystectomy outcomes^1^. The cohort has already been described extensively^1,12^. The study was approved by the Regional Research Ethics Committee in Uppsala, Sweden (2017/022, 2019-06128, 2021-05273). The manuscript was structured in adherence with the STROBE checklist for cohort studies^13^. Throughout the manuscript, gender refers to surgeons, as this variable was inferred indirectly from names and relates to behavioural factors, whereas sex refers to patients’ biological sex.

### Setting and population

As in the previous study, all open and laparoscopic cholecystectomies registered in GallRiks between 1 January 2007 and 31 December 2019 were included. Cholecystectomies performed for malignant indications and cases with incomplete surgeon gender data were excluded. The GallRiks registry contains data on patient demographics, surgery-specific variables, and intraoperative complications, with a 30-day follow-up period to capture postoperative adverse events. The organization, structure, and reliability of the GallRiks registry have been described elsewhere^14^. Participation is predicated on a waiver of consent, wherein patients are informed in writing about their inclusion before surgery. Patients can decline participation and request to access, amend, or remove their data at their discretion^15^. Data withdrawal is rare. Surgeon gender is not recorded in the registry but was inferred from names and added to the data set by the registry holder. Sweden’s small surgical community enabled verification of ambiguous cases.

### Main outcomes and measures

The primary outcome was the occurrence of surgical complications, including significant blood loss (defined as bleeding requiring advanced intervention, blood transfusion, or conversion to open surgery), bile duct injury, visceral organ injury, postoperative bile leakage, and postoperative abscesses. Bile duct injuries were analysed separately. Secondary outcomes included total complications within 30 days of surgery, consisting of all intraoperative and postoperative complications, such as surgical complications, pulmonary and cardiac complications, thrombosis, wound infections, and urinary tract infections. Additional secondary outcomes were hospital stay > 3 days and operating time. The primary exposure variable was surgeon gender, with outcomes assessed according to surgeon caseload, case mix, team composition, and the surgeon–patient gender relationship. Team composition was defined by the lead and assistant surgeons, who could be residents, but did not include anaesthetists or nursing staff.

### Potential confounders

All models were adjusted for patient characteristics, including sex, age, American Society of Anaesthesiologists (ASA) fitness grade, treatment year, hospital type (university, regional, county, or private) and the presence of acute cholecystitis or a history of cholecystitis. Surgeon characteristics included caseload by experience at the time of each procedure (< 3, 3–8, or > 8 years) and the surgeon’s annual volume in the preceding year, calculated as the number of cholecystectomies performed in the 12 months before the operation date. In analyses specifically focusing on surgeon caseload, experience in years and total number of cholecystectomies performed (< 50, 50–150, or > 150 cholecystectomies) were considered, excluding annual volume as a separate variable. The cut-off points for caseload categories were determined to ensure approximately equal group sizes. Case-mix analyses incorporated additional covariates, including body mass index (BMI) and patient co-morbidities such as diabetes, chronic obstructive pulmonary disease (COPD), cardiovascular disease (CVD), bleeding disorders and/or ongoing anticoagulant therapy, and immunosuppression. These analyses were restricted to the 2017–2019 period, as most co-morbidity data were available in GallRiks only from 2017 onwards. In the full cohort, BMI and co-morbidities were partially accounted for using the ASA physical status classification.

### Statistical analysis

Demographic characteristics of patients in the full cohort and the 2017–2019 subcohort were summarized in contingency tables. Continuous variables are reported as means with mean differences and 95% confidence intervals, whereas categorical variables are reported as differences in proportions with 95% confidence intervals. The associations between surgeon gender and surgical complications, bile duct injury, total complications, and hospital stay > 3 days were analysed using binomial logit models, with cluster-robust standard errors at the clinic level to account for dependencies (sandwich version 3.1.1 in R version 4.4.1; R Foundation for Statistical Computing, Vienna, Austria)^16^. Categorical covariates were dummy-coded, whereas non-constant associations between other covariates and outcomes were accommodated using restricted cubic splines, with inner knots placed at the 33rd and 66th empirical percentiles. Missing data in dichotomous outcome analyses were handled using multiple imputations by chained equations, generating 20 data sets using an imputation model that mirrored the analysis model combined with weight and height as auxiliary variables for BMI (mice version 3.16.0 in R version 4.4.1)^17^. The variables subjected to imputation and their corresponding missingness rates are shown in *Table S1*. For models examining caseload, team composition (including gender of the surgeon and assistant surgeon, if applicable), and surgeon–patient gender configuration, the complete 2007–2019 cohort was used as it had minimal missing data. The analyses of case mix were restricted to the 2017–2019 period, because the variables were introduced in 2017. Marginal point estimates were derived by marginal standardization with 95% confidence intervals using the delta method (marginal effects version 0.22.0 in R version 4.4.1)^18,19^. A log transformation was applied to operating times before analysis using linear mixed-effects models to address the positively skewed distribution of operating times. These models incorporated random intercepts for surgeons nested within clinics (nlme version 3.1.64 in R version 4.4.1)^20^. For each surgeon gender and subgroup combination, operating times are reported as geometric means, with corresponding ratios provided to facilitate intergroup comparison. For the other outcomes, results are presented as surgeon gender- and subgroup-specific risk percentages, accompanied by corresponding risk ratios (RRs) with 95% confidence intervals. Lorenz curves were used to plot the distribution of surgical complications and bile duct injuries across cholecystectomy volume groups (< 50, 50–150, or > 150). Two-sided *P* < 0.050 was considered significant.

## Results

**Fig. 1:**
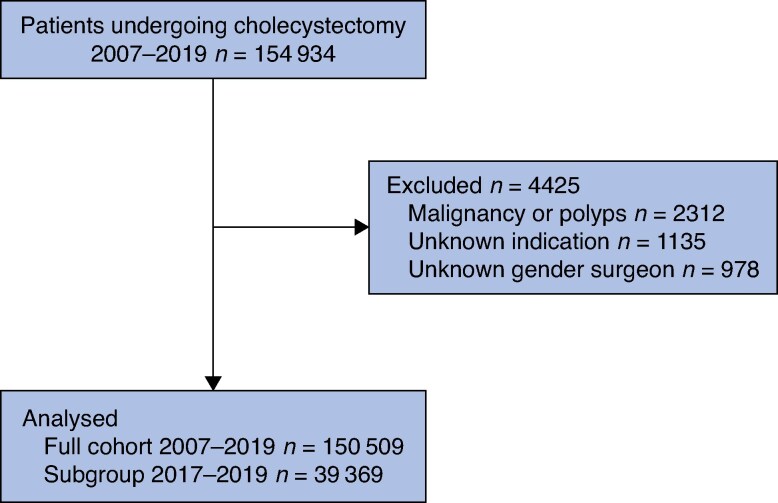
Study flow chart

The final analysis included 150 509 cholecystectomies, with 39 369 operations (26.2%) performed in 2017–2019 (*Fig. 1*). The procedures were performed by 2555 surgeons, of whom 849 (33.2%) were female and 1706 (66.8%) male. The 2017–2019 subgroup comprised 1472 surgeons, of whom 504 (34.2%) were female and 968 (65.8%) male. *Table 1* shows patient demographics and the extent of missing data. In the full cohort, complication rates were lower among patients operated on by female surgeons compared with male surgeons, consistent with previously published findings^1^. Detailed outcome measures, including bile duct injuries, total complication rates, length of hospital stay, and operating time, have been reported previously^1^.

**Table 1:** Demographics of study patients

	All patients, 2007–2019			Case-mix cohort, 2017–2019		
	Female surgeon (*n* = 37 847)	Male surgeon (*n* = 112 662)	Difference (%)*	Female surgeon (*n* = 11 566)	Male surgeon (*n* = 27 803)	Difference (%)*
**Age (years)**						
Mean(s.d.)	58.0(16.5)	59.3(16.7)	−1.3 (−1.5, −1.2)	55.0(16.7)	55.7(16.6)	−0.7 (−1.1, −0.3)
Median (i.q.r.)	58 (45–71)	59 (47–73)		55 (42–69)	56 (43–69)	
Missing	79	251		17	50	
**Patient sex**						
Male	12 271 (32.4%)	39 030 (34.7%)	−2.2 (−2.8, −1.7)	4002 (34.6%)	10 219 (36.8%)	−2.1 (−3.2, −1.1)
Female	25 568 (67.6%)	73 607 (65.3%)	2.2 (1.7, 2.8)	7562 (65.4%)	17 583 (63.2%)	2.1 (1.1, 3.2)
Missing	8	25		2	1	
**ASA fitness grade**						
I	17 090 (45.3%)	53 389 (47.6%)	−2.3 (−2.9, −1.7)	4372 (37.9%)	10 720 (38.7%)	−0.8 (−1.8, 0.3)
II–III	17 352 (46.0%)	49 035 (43.7%)	2.3 (1.7, 2.9)	5900 (51.2%)	13 817 (49.9%)	1.3 (0.2, 2.4)
≥ IV	3314 (8.8%)	9853 (8.8%)	0.0 (−0.3, 0.3)	1259 (10.9%)	3170 (11.4%)	−0.5 (−1.2, 0.2)
Missing	91	385		35	96	
**BMI (kg/m^2^)**†						
Mean(s.d.)	n.a.	n.a.		28.6(5.2)	28.8(5.4)	−0.2 (−0.3, −0.1)
Median (i.q.r.)	n.a.	n.a.		28 (25–32)	28 (25–32)	
Missing	n.a.	n.a.		2209	6587	
**Patient co-morbidities**†						
Diabetes	n.a.	n.a.		846 (7.4%)	2016 (7.3%)	0.1 (−0.5, 0.6)
Missing	n.a.	n.a.		112	341	
Cardiovascular disease	n.a.	n.a.		2094 (18.3%)	4935 (18.0%)	0.3 (−0.6, 1.1)
Missing	n.a.	n.a.		118	373	
COPD	n.a.	n.a.		855 (7.5%)	1879 (6.9%)	0.6 (0.1, 1.2)
Missing	n.a.	n.a.		136	385	
Bleeding	n.a.	n.a.		550 (4.8%)	1415 (5.2%)	−0.4 (−0.8, 0.1)
Missing	n.a.	n.a.		131	381	
Immunosuppression	n.a.	n.a.		261 (2.3%)	586 (2.1%)	0.1 (−0.2, 0.5)
Missing	n.a.	n.a.		122	377	
**Setting**						
Acute	12 667 (33.5%)	40 087 (35.6%)	−2.1 (−2.7, −1.6)	6923 (59.9%)	16 292 (58.6%)	1.3 (0.2, 2.3)
Elective	25 180 (66.5%)	72 575 (64.4%)	2.1 (1.6, 2.7)	4643 (40.1%)	11 511 (41.4%)	−1.3 (−2.3, −0.2)
Ongoing acute cholecystitis	7246 (19.1%)	24 313 (21.6%)	−2.4 (−2.9, −2.0)	2697 (23.3%)	6965 (25.1%)	−1.7 (−2.7, −0.8)
History of acute cholecystitis	4317 (11.4%)	13 980 (12.4%)	−1.0 (−1.4, −0.6)	1361 (11.8%)	3491 (12.6%)	−0.8 (−1.5, −0.1)
**Hospital type**						
University hospital	10 197 (26.9%)	25 324 (22.5%)	4.5 (4.0, 5.0)	3167 (27.4%)	6189 (22.3%)	5.1 (4.2, 6.1)
Regional hospital	12 053 (31.8%)	39 435 (35.0%)	−3.2 (−3.7, −2.6)	3627 (31.4%)	9736 (35.0%)	−3.7 (−4.7, −2.6)
County hospital	11 148 (29.5%)	38 146 (33.9%)	−4.4 (−4.9, −3.9)	3652 (31.6%)	8580 (30.9%)	0.7 (−0.3, 1.7)
Private clinic	4449 (11.8%)	9757 (8.7%)	3.1 (2.7, 3.5)	1120 (9.7%)	3298 (11.9%)	−2.2 (−2.8, −1.5)

Values are *n* (%) unless otherwise stated; *values in parentheses are 95% confidence intervals. †Only available in the subgroup 2017–2019. s.d., Standard deviation; i.q.r., interquartile range; ASA, American Society of Anesthesiologists; BMI, body mass index; n.a., not available; COPD, chronic obstructive pulmonary disease.

### Main results

#### Surgeon gender and caseload

**Fig. 2:**
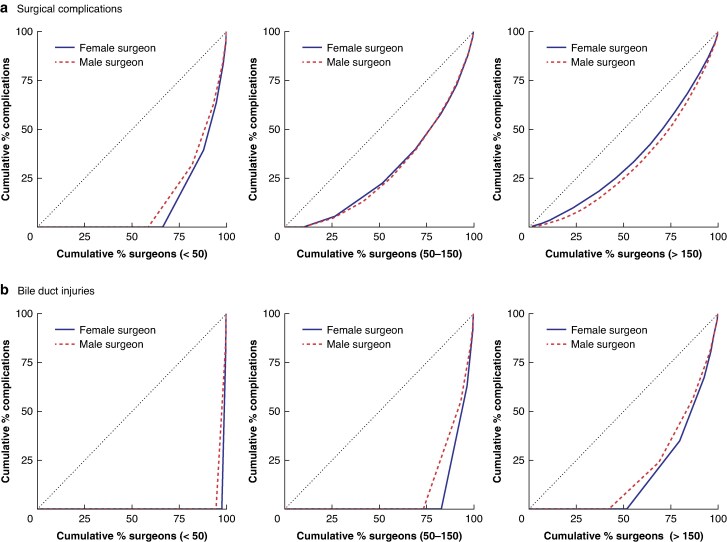
Lorenz curves illustrating the distribution of surgical complications and bile duct injuries across different cholecystectomy volume groups: < 50, 50−150, and > 150

Female surgeons had lower surgical volumes of cholecystectomies, with high-volume surgeons being predominantly male (*Table S2*). Covariate adjustments for surgeon experience (< 3, 3–8, or > 8 years) and cumulative volume (< 50, 50–150, or > 150 cholecystectomies) showed fewer surgical complications for female surgeons across all experience levels (*Table 2*). This disparity grew with experience. Male surgeons with > 8 years of experience had a 50% increased risk of surgical complications compared with their female counterparts. Although the risk of bile duct injury increased with surgical experience, a significant gender difference was evident only in surgeons with < 3 years of experience (RR 1.77, 95% confidence interval 1.30 to 2.40) or performing < 50 cholecystectomies (RR 1.55, 1.18 to 2.03). Male surgeons had a marginally higher risk of total complications in some volume groups (*Table 2*). Patients operated on by inexperienced male surgeons (< 3 years’ experience) had a higher risk of a prolonged hospital stay. Across both genders, operating time decreased with accumulating experience (*Table 2*). Nevertheless, female surgeons consistently recorded slightly longer operating times, a trend most pronounced among highly experienced practitioners. When plotting the distribution of surgical volume and complications at the individual level using Lorenz curves, a higher proportion of female surgeons performing < 50 cholecystectomies had never caused a surgical complication (*Fig. 2*). In higher-volume groups, all surgeons had at least one complication. Notably, a higher proportion of female surgeons had no history of bile duct injuries, regardless of surgical volume.

**Table 2:** Surgeon gender–outcome relationship and surgeon experience and volume of cholecystectomies, 2007–2019

Outcome	Years of experience/volume		Male surgeon	Female surgeon	Difference	*P*
			Risk (%)*	Risk (%)*	Risk ratio*	
						
Surgical complications§	Years of experience	< 3	3.78 (3.49, 4.07)	3.37 (2.87, 3.87)	1.12 (0.99, 1.27)	0.076
		3–8	4.38 (4.01, 4.75)	3.57 (3.09, 4.05)	1.23 (1.07, 1.41)	0.004
		> 8	5.05 (4.59, 5.50)	3.37 (2.78, 3.96)	1.50 (1.23, 1.82)	<0.001
	Cumulative volume	< 50	3.91 (3.62, 4.20)	3.45 (3.00, 3.91)	1.13 (1.01, 1.28)	0.039
		50–150	4.64 (4.26, 5.03)	3.66 (3.23, 4.08)	1.27 (1.13, 1.43)	<0.001
		> 150	4.28 (3.87, 4.70)	3.02 (2.22, 3.81)	1.42 (1.10, 1.83)	0.007
Bile duct injury§	Years of experience	< 3	0.32 (0.27, 0.39)	0.19 (0.14, 0.24)	1.77 (1.30, 2.40)	<0.001
		3–8	0.38 (0.31, 0.45)	0.32 (0.22, 0.43)	1.17 (0.82, 1.67)	0.387
		> 8	0.52 (0.42, 0.62)	0.32 (0.15, 0.50)	1.59 (0.96, 2.64)	0.073
	Cumulative volume	< 50	3.53 (2.84, 4.22)	2.28 (1.69, 2.87)	1.55 (1.18, 2.03)	0.002
		50–150	4.28 (3.64, 4.93)	2.79 (1.59, 3.99)	1.54 (0.96, 2.45)	0.072
		> 150	3.78 (2.83, 4.74)	3.56 (1.78, 5.34)	1.06 (0.66, 1.72)	0.803
Total complications§	Years of experience	< 3	9.56 (8.85, 10.25)	8.92 (8.13, 9.70)	1.07 (0.99, 1.15)	0.065
		3–8	10.71 (9.86, 11.56)	9.79 (8.97, 10.62)	1.09 (1.02, 1.18)	0.019
		> 8	11.19 (10.27, 12.11)	9.94 (8.79, 11.09)	1.13 (1.01, 1.25)	0.032
	Cumulative volume	< 50	9.87 (9.17, 10.57)	9.28 (8.51, 10.04)	1.06 (0.99, 1.14)	0.096
		50–150	11.01 (10.19, 11.82)	9.86 (9.01, 10.71)	1.12 (1.04, 1.20)	0.004
		> 150	10.10 (9.21, 10.99)	9.22 (7.82, 10.62)	1.10 (0.97, 1.24)	0.154
Hospital stay > 3 days§	Years of experience	< 3	10.59 (9.64, 11.55)	9.36 (8.37, 10.35)	1.13 (1.05, 1.22)	0.002
		3–8	11.80 (10.59, 13.02)	11.07 (9.65, 12.49)	1.07 (0.98, 1.16)	0.066
		> 8	11.58 (10.07, 13.08)	9.91 (8.13, 11.68)	1.17 (1.00, 1.36)	0.047
	Cumulative volume	< 50	11.58 (10.60, 12.56)	10.37 (9.34, 11.39)	1.12 (1.06, 1, 18)	<0.001
		50–150	12.00 (10.73, 13.27)	10.72 (9.52, 11.91)	1.12 (1.02, 1.23)	0.014
		> 150	9.24 (7.81, 10.67)	9.44 (6.84, 12.03)	0.98 (0.77, 1.25)	0.865
Operating time (minutes)§	Years of experience	< 3	90.69 (87.91, 93.56)†	96.85 (93.75, 100.00)†	0.94 (0.92, 0.95)‡	<0.001
		3–8	87.06 (84.32, 89.89)†	94.40 (91.25, 97.66)†	0.92 (0.91, 0.94)‡	<0.001
		> 8	81.33 (78.74, 84.01)†	89.65 (86.52, 92.89)†	0.91 (0.89, 0.93)‡	<0.001
	Cumulative volume	< 50	95.60 (92.59, 98.71)†	103.20 (99.80, 106.70)†	0.93 (0.91, 0.94)‡	<0.001
		50–150	86.41 (83.68, 89.23)†	94.12 (90.94, 97.42)†	0.92 (0.90, 0.93)‡	<0.001
		> 150	73.13 (70.71, 75.63)†	81.24 (78.23, 84.38)†	0.90 (0.88, 0.92)‡	<0.001

Values in parentheses are 95% confidence intervals. *Unless otherwise indicated; values are †geometric means and ‡ operating time ratios. §Adjusted for patient age, sex, American Society of Anesthesiologists fitness grade, ongoing acute cholecystitis, history of acute cholecystitis, treatment year, hospital type, surgeon years of experience, and cumulative operative volume of cholecystectomies. Associations between surgeon gender and surgical complications, bile duct injury, and hospital stay were analysed using binomial logic models, whereas operating time was analysed using linear mixed-effects models.

#### Surgeon gender and case mix

Controlling for case mix, including age, sex, ASA grade, BMI, acute cholecystitis (current and historical), and co-morbidities (diabetes, chronic obstructive pulmonary disease, cardiovascular disease, bleeding disorders, ongoing anticoagulant therapy, and immunosuppression), male surgeons exhibited significantly increased rates of surgical complications, bile duct injuries, and total complications, compared with female surgeons. Their patients also experienced longer hospital stays, despite having slightly shorter operating times (*Table 3*).

**Table 3:** Surgeon gender–outcome relationship and patient case mix, 2017–2019

Outcome	Male surgeon		Female surgeon		Difference	*P*
	*n* (%)	Risk (%)*†	*n* (%)	Risk (%)*†	Risk ratio†	
Surgical complications¶	1189 (4.3%)	4.22 (3.92, 4.54)	372 (3.2%)	3.31 (2.89, 3.73)	1.28 (1.12, 1.45)	<0.001
Bile duct injury¶	111 (0.4%)	0.39 (0.31, 0.47)	26 (0.2%)	0.24 (0.13, 0.34)	1.63 (1.01,2.62)	0.045
Total complications¶	2870 (10.4%)	10.31 (9.38, 11.23)	1057 (9.2%)	9.34 (8.44, 10.23)	1.10 (1.03, 1.19)	0.008
Hospital stay > 3 days¶	2734 (9.9%)	9.84 (8.62, 11.05)	960 (8.3%)	8.46 (7.25, 9.67)	1.16 (1.04, 1.30)	0.010
Operating time (minutes)¶		89.64 (86.45, 92.96)‡		97.44 (93.76, 101.30)‡	0.92 (0.90, 0.94)§	<0.001

Values are *n* (%) unless otherwise stated; *values in parentheses are 95% confidence intervals. †Unless otherwise indicated; values are ‡geometric means and §operating time ratios. ¶Adjusted for patient age, sex, American Society of Anesthesiologists classification, ongoing acute cholecystitis, history of acute cholecystitis, treatment year, hospital type, surgeon years of experience, surgeon annual volume, body mass index, diabetes, chronic obstructive pulmonary disease; cardiovascular disease, immunosuppression, and increased risk of bleeding. Associations between surgeon gender and surgical complications, bile duct injury, and hospital stay were analysed using binomial logic models, whereas operating time was analysed using linear mixed-effects models.

#### Surgeon–team gender composition

Among operations performed by male surgeons, 66 890 (59.4%) had no registered assisting surgeon, 13 263 (11.8%) had a female surgeon as assistant, and 32 509 (28.9%) had a male surgeon. The corresponding figures for operations performed by female surgeons were 16 623 (43.9%) with no assistant, 5566 (14.7%) with a female assistant, and 15 658 (41.4%) with a male assistant. Analysing outcomes based on team composition (lead and assisting surgeon), all-male teams had the highest surgical complication risk, whereas all-female teams had a significantly lower risk (*Table 4*). A comparable pattern was observed in bile duct injury for teams composed entirely of women and for teams of exclusively men. These trends persisted across total complications and length of hospital stay, with female-led teams achieving superior outcomes. Female surgeons operating alone had the lowest complication risk. Operating times were longer for female lead surgeons across all configurations (*Table 4*).

**Table 4:** Surgeon gender–outcome relationship and team composition, 2007–2019

Outcome	Assistant gender	Male surgeon	Female surgeon	Difference	*P*
		Risk (%)*	Risk (%)*	Risk ratio*	
Surgical complications§	None	3.42 (3.18, 3.66)	2.50 (2.15, 2.84)	1.37 (1.21, 1.55)	<0.001
	Male	5.46 (4.99, 5.94)	4.45 (3.89, 5.01)	1.23 (1.10, 1.37)	<0.001
	Female	5.08 (4.54, 5.61)	3.93 (3.19, 4.73)	1.28 (1.07, 1.52)	0.003
Bile duct injury§	None	0.20 (0.16, 0.24)	0.13 (0.07, 0.18	1.55 (0.98, 2.45)	0.058
	Male	0.70 (0.60, 0.81)	0.41 (0.29, 0.53)	1.71 (1.27, 2.29)	<0.001
	Female	0.50 (0.39, 0.62)	0.30 (0.11, 0.49)	1.65 (0.83, 3.31)	0.156
Total complications§	None	9.01 (8.38, 9.64)	8.02 (7.22, 8.82)	1.12 (1.03, 1.22)	0.005
	Male	12.07 (11.02, 13.12)	11.07 (10.18, 11.95)	1.09 (1.02, 1.17)	0.017
	Female	11.97 (10.99, 12.94)	10.00 (9.05, 10.95)	1.20 (1.09, 1.31)	<0.001
Hospital stay > 3 days§	None	8.54 (7.63, 9.46)	7.40 (6.48, 8.33)	1.15 (1.06, 1.25)	<0.001
	Male	14.98 (13.59, 16.36)	13.00 (11.70, 14.29)	1.15 (1.07, 1.24)	<0.001
	Female	14.68 (13.49, 15.86)	12.11 (10.38, 13.84)	1.21 (1.08, 1.36)	<0.001
Operating time (minutes)§	None	78.92 (76.46, 81.45)†	86.80 (83.95, 89.74)†	0.91 (0.90, 0.92)‡	<0.001
	Male	99.43 (96.30, 102.70)†	104.40 (101.00, 107.90)†	0.95 (0.94, 0.97)‡	<0.001
	Female	101.80 (98.51, 105.20)†	107.90 (104.20, 111.70)†	0.94 (0.93, 0.96)‡	<0.001

Values in parentheses are 95% confidence intervals. *Unless otherwise indicated; values are †geometric means and ‡ operating time ratios. §Adjusted for patient age, sex, American Society of Anesthesiologists fitness grade, ongoing acute cholecystitis, history of acute cholecystitis, treatment year, hospital type, surgeon years of experience, and annual volume. Associations between surgeon gender and surgical complications, bile duct injury, and hospital stay were analysed using binomial logic models, whereas operating time was analysed using linear mixed-effects models.

#### Surgeon–patient gender composition

An analysis of surgeon–patient gender interactions showed that women operated on by female surgeons had the lowest risk for all outcomes. Consistent with established cholecystectomy risk factors^21^, male patients systematically had a higher risk of complications (*Table 5*). However, women operated on by male surgeons had a higher risk of surgical complications and bile duct injuries. An increase in the risk of surgical complications was also observed among men treated by male surgeons; however, no statistically significant difference was detected in rates of bile duct injury. For total complications, both male and female patients had an increased risk with male surgeons. This trend was also observed for extended hospital stays (*Table 5*). Examination of team composition and patient sex showed that all-female surgical teams yielded superior outcomes for female patients (data not shown).

**Table 5:** Surgeon gender–outcome relationship and patient sex, 2007–2019

Outcome	Patient sex	Male surgeon	Female surgeon	Difference	*P*
		Risk (%)*	Risk (%)*	Risk ratio*	
Surgical complications§	Male	5.34 (4.97, 5.70)	4.43 (3.90, 4.95)	1.21 (1.10, 1.33)	<0.001
	Female	3.67 (3.39, 3.96)	2.92 (2.58, 3.27)	1.26 (1.13, 1.40)	<0.001
Bile duct injury§	Male	0.46 (0.38.0.53)	0.37 (0.24, 0.49)	1.26 (0.89, 1.76)	0.190
	Female	0.35 (0.29, 0.40)	0.20 (0.14, 0.27)	1.69 (1.20, 2.39)	0.003
Total complications§	Male	12.46 (11.55, 13.36)	11.56 (10.65, 12.46)	1.08 (1.01, 1.15)	0.028
	Female	9.19 (8.59, 9.79)	8.32 (7.69, 8.95)	1.10 (1.04, 1.18)	0.002
Hospital stay > 3 days§	Male	15.10 (13.73, 16.46)	13.59 (12.18, 15.00)	1.11 (1.04, 1.18)	0.002
	Female	9.27 (8.36, 10.19)	8.25 (7.34, 9.16)	1.12 (1.04, 1.21)	0.001
Operating time (minutes)§	Male	95.48 (92.47, 98.60)†	103.9 (100.50, 107.50)†	0.92 (0.90, 0.93)‡	<0.001
	Female	83.45 (80.88, 86.10)†	91.04 (88.11, 94.07)†	0.92 (0.90, 0.93)‡	<0.001

Values in parentheses are 95% confidence intervals. *Unless otherwise indicated; values are †geometric means and ‡ operating time ratios. §Adjusted for patient age, sex, American Society of Anesthesiologists fitness grade, ongoing acute cholecystitis, history of acute cholecystitis, treatment year, hospital type, surgeon's years of experience, and annual volume. Associations between surgeon gender and surgical complications, bile duct injury, and hospital stay were analysed using binomial logic models, whereas operating time was analysed using linear mixed-effects models.

## Discussion

This large population-based cohort study has demonstrated that differences in outcomes between female and male surgeons are unlikely to be explained by caseload, case mix, team composition, or the surgeon–patient relationship. Female surgeons consistently had better outcomes, including reduced rates of surgical complications, bile duct injuries, and total complications, and shorter hospital stays. Male surgeons with limited experience had a significantly increased risk of bile duct injury, highlighting the importance of rigorous mentorship during early years of practice. All-male teams had the highest complication rates. Women operated on by female surgeons had the lowest complication rates. These findings strengthen the previously reported association between surgeon gender and clinical outcomes^1^.

The association between surgeon gender and patient outcomes remains a matter of debate. Previous research^12,22^ demonstrated a positive association between surgeon volume and improved cholecystectomy outcomes. When accounting for caseload, male surgeons consistently exhibited higher complication rates, especially in high-volume groups. Bile duct injury rates were higher among the most experienced surgeons, likely reflecting their management of more complex cases or being consulted in the event of an injury. Nonetheless, statistically significant gender differences were observed only in the less experienced cohort. These differences may suggest a greater propensity for risk-taking among younger male surgeons, as reported in other settings such as trauma^23,24^. Additionally, although operating time decreased with experience for both genders, female surgeons consistently had marginally longer operating times, possibly reflecting a more cautious surgical approach throughout their careers. This finding is supported by the higher proportion of female surgeons with no record of bile duct injuries, regardless of surgical volume. It has been hypothesized that the longer operating times for female surgeons might result from receiving less assistance from nurse assistants who could be uncomfortable being led by women^25,26^. However, this is unlikely in the Swedish context, where female surgeons are relatively common, nursing staff are accustomed to working under female surgical leadership, and most surgical units use prearranged decks containing all the necessary equipment for cholecystectomy and cholangiography, ensuring that workflow is not hindered by missing instruments. Shorter operating times may suggest greater efficiency, but they can also reflect pressure to increase productivity and decrease costs. However, excessively rapid operations have been linked to higher complication rates^27^.

Despite adjustments for case mix being standard practice, this remains one of the most frequently cited counterarguments regarding gender-related outcome disparities^8^. This underscores the importance of the extended analysis presented, which reaffirms that differences in outcomes cannot be fully explained by case mix alone. These findings are consistent with those of previous studies^2,4,28^ that challenge the case-mix hypothesis. For example Okoshi *et al*.^3^ reported that female Japanese gastrointestinal surgeons managed more complex cases and performed more open procedures, yet attained comparable outcomes to their male counterparts. Similarly, a recent Swedish study^2^ on emergency colon cancer resections reported better patient outcomes for female surgeons, including fewer severe postoperative complications, less need for intensive care, reduced reoperations, and improved long-term survival. The authors discussed case mix but found no evidence of impact. The results suggested that intrinsic differences in surgical practice and team dynamics, rather than case complexity, may explain the observed disparities.

Another explanation is that female surgeons may defer complex cases or complications to a more senior male colleague, who is recorded as the lead surgeon. However, the present study contradicts this hypothesis, as teams led by male surgeons consistently had worse outcomes, with all-male teams exhibiting the highest complication rates. Optimal outcomes were achieved in procedures without an assistant, likely reflecting simpler procedures performed under ideal conditions. Although studies of anaesthetist–surgeon gender combinations have shown no significant effects of these on complications or mortality^29,30^, the findings align with research indicating an association between all-male cardiac surgical teams and longer hospital stays^9^.

The present results indicate that women achieved optimal outcomes when operated on by female surgeons. This finding is consistent with research showing higher complication and mortality rates in women treated by male surgeons^10,11^. In contrast, male patients do not display a similar disparity. In the present analysis, men also showed improved outcomes when treated by female surgeons. Gender discordance between surgeons and patients has been associated with worse outcomes in complex cancer operations^28^. Similarly, lower mortality rates and fewer readmissions have been reported among older women with emergency medical conditions treated by female physicians^31^. These differences are often attributed to variations in communication styles and decision-making processes. Male physicians may underestimate the severity of illness or pain in women, potentially resulting in delayed or suboptimal care^32,33^. Further research is needed to determine the precise impact of gender discordance on intraoperative complications.

This main strength of this study is the use of a large, nationally representative database, with 94% national coverage in 2023^34^. The registry’s ability to capture serious adverse events has been validated^35^. Focusing on a single procedure enhances the robustness of the present findings. However, several limitations should be acknowledged. Residual confounding may persist owing to unmeasured case-mix variables and the absence of direct measures of surgical complexity. Surgeon gender was inferred from names, which could have introduced exposure misclassification. Additionally, data on surgeons’ experience before the registry’s inception were not available. Although the lead surgeon is defined as the individual performing most of the cholecystectomy, in the event of complications, a consultant surgeon may have been documented as the primary surgeon, which could have affected outcome attribution. Missing data were minimal in the cohort, and the results remained consistent with those of the dichotomous models. Nevertheless, the imputation of missing values could have introduced some degree of uncertainty. The study included use of multiple models to thoroughly assess each outcome and relevant interactions. However, this may have introduced a greater likelihood of type I error, and the results should be interpreted with appropriate caution. The cohort included data from 2007–2019 and no major changes in cholecystectomy techniques occurred in Sweden during this period. However, the proportion of acute procedures has increased since the COVID-19 pandemic^34^. As of 2025, surgical techniques remain consistent and robotic surgery is uncommon, minimizing concerns about procedural advancements affecting the findings. Furthermore, the registry does not include data on surgeon characteristics, such as age, specific technical skills, or experience in other specialties, which would enrich interpretation of the observed outcomes. The data for this study were derived from a national sample in Sweden, with a publicly funded healthcare system that does not allow patients to select their surgeon. Consequently, the findings may have limited generalizability to countries with varying healthcare structures or patient selection processes.

Several factors may explain gender differences in surgical outcomes, including female physicians’ emphasis on patient-centred communication and adherence to guidelines^36–38^. However, nuances in surgical technique, teamwork, and decision-making may also play a role^1^. A Swedish study^39^ of the personality traits of general surgeons found that female surgeons scored more highly in conscientiousness and neuroticism, traits that may hypothetically be associated with enhanced precision and improved patient outcomes, notwithstanding longer operating times. Further research is warranted to explore the impact of institutional and environmental influences, including mentorship, team dynamics, and training, on outcomes.

A key observation from this study is the higher rate of bile duct injuries in patients operated on by inexperienced male surgeons. This finding may reflect variability in early-career risk assessment, decision-making, and skill acquisition, underscoring the importance of mentorship and structured support during the formative years of surgical practice. Simulation-based surgical training offers demonstrable benefits; studies^40,41^ have indicated a 30% increase in operating speed and a six-fold reduction in errors among simulator-trained residents compared with their non-simulator-trained peers. Furthermore, it is essential to emphasize prioritization of surgical quality over speed, particularly for younger surgeons who may encounter implicit or explicit pressures to operate quickly.

Although gender appears to be associated with differences in outcomes, it is likely a proxy for underlying behaviours or attitudes, such as thoroughness, caution, and communication, which may be more prevalent in one group but are not exclusive to it. Adopting practices more commonly used by female surgeons, such as enhanced communication, collaboration, and a greater focus on patient-centred care, may improve surgical outcomes^36,37^. The challenges female surgeons face in male-dominated fields may lead to selection of a particularly skilled cohort^6,42^, underlining the importance of determining their identifying characteristics to optimize surgical education and training for all genders. Ongoing learning, mentorship, and fostering collaborative diverse team dynamics are essential for improving patient outcomes.

Overall, surgical complication rates following cholecystectomies in Sweden are low. Nonetheless, continuous efforts to improve surgical safety remain essential. Guidelines such as the Society of American Gastrointestinal and Endoscopic Surgeons recommendations for achieving a critical view of safety and implementing a brief pause before dividing major structures^43,44^ support these findings, and emphasize the importance of prioritizing caution over speed to enhance surgical safety, regardless of surgeon gender.

## Supplementary Material

zraf179_Supplementary_Data

## Data Availability

The deidentified participant data and statistical code are available from the corresponding author with publication upon reasonable request and after the research team has approved the proposal.
