# Supplementary material for: Impact of surgeon caseload, case mix, and team composition on gender disparities in cholecystectomy outcomes
Source: BJS Open. 2026 Mar 27;10(2):zraf179. doi: 10.1093/bjsopen/zraf179 (PMC13023730; doi:10.1093/bjsopen/zraf179)
Supplement: zraf179_Supplementary_Data [file zraf179_supplementary_data.docx]

**Supplementary Material**

Title: Impact of surgeon caseload, case mix and team composition on gender disparities in cholecystectomy outcomes

**Authors:**

My Blohm^1,2,3^, Riccardo LoMartire^3,4^, Gabriel Sandblom^1^, Lars Enochsson^5,6^, Johanna Österberg ^1,2,3^

**Affiliations:**

^1^ Department of Clinical Science and Education, South General Hospital, Karolinska Institutet, Stockholm, Sweden

^2^ Department of Surgery, Mora Hospital, Mora, Sweden

^3^ Centre for Clinical Research Dalarna, Uppsala University, Falun, Sweden

^4^ School of Health and Welfare, Dalarna University, Falun, Sweden

^5^ Department of Diagnostics and Intervention, Surgery, Umeå University, Umeå, Sweden

^6^ Department of Clinical Science, Intervention and Technology, Division of Orthopaedics and Biotechnology, Karolinska Institutet, Stockholm, Sweden

**Corresponding author:**

My Blohm

Department of Surgery, Mora Hospital, S-792 85 Mora, Sweden

[my.blohm@ki.se](mailto:my.blohm@ki.se)

ORCID ID: 0000-0002-0457-5165

**Supplementary Materials - Index**

| **Supplementary Tables** |  |
| --- | --- |
| Supplementary Table 1 | *page 3* |
| Supplementary Table 2 | *page 4* |
|  |  |
|  |  |
|  |  |

| **Supplementary table 1. Proportion of missing data for each imputed variable** | | | | | | |
| --- | --- | --- | --- | --- | --- | --- |
|  | **All surgeons** | | **Male surgeon** | | **Female surgeon** | |
|  | Frequency* | Percent | Frequency* | Percent | Frequency* | Percent |
| **Surgeon years of experience** | 4 | 0 | 1 | 0.0 | 2 | 0.0 |
| **Patient related variables** |  |  |  |  |  |  |
| Sex | 3 | 0 | 2 | 0.0 | 2 | 0.0 |
| Age | 67 | 0.2 | 6 | 0.0 | 3 | 0.0 |
| ASA grade | 131 | 0.3 | 50 | 0.2 | 17 | 0.1 |
| Diabetes | 453 | 1.2 | 341 | 1.2 | 112 | 1.0 |
| CVD | 491 | 1.2 | 373 | 1.3 | 118 | 1.0 |
| Immunosuppression | 499 | 1.3 | 377 | 1.4 | 122 | 1.1 |
| COPD | 521 | 1.3 | 381 | 1.4 | 131 | 1.1 |
| Bleeding | 512 | 1.3 | 385 | 1.4 | 136 | 1.2 |
| Height | 6772 | 17.2 | 5184 | 18.6 | 1588 | 13.7 |
| Weight | 6187 | 15.7 | 4824 | 17.4 | 1363 | 11.8 |
| Body mass index | 8796 | 22.3 | 6587 | 23.7 | 2209 | 19.1 |
| **Outcomes** |  |  |  |  |  |  |
| Operating time | 9 | 0 | 96 | 0.3 | 35 | 0.3 |
| Total complications | 278 | 0.7 | 153 | 0.6 | 67 | 0.6 |
| Hospital stay >3 days | 220 | 0.6 | 196 | 0.7 | 82 | 0.7 |

**Footnote: * frequency of missing data**

| **Supplementary Table 2. Distribution of procedures performed by female and male surgeons in 2007 and 2019** | | | | |  | |
| --- | --- | --- | --- | --- | --- | --- |
|  | **2007** | | **2019** | | |  |
|  | **Female surgeon**  **(n=1,591)** | **Male surgeon**  **(n=6,904)** | **Female surgeon**  **(n=4,066)** | **Male surgeon**  **(n=9,695)** | |  |
| **Hospital type, n (%)** |  |  |  |  | |  |
| University hospital | 386 (24.3) | 1,761 (25.5) | 1,089 (26.8) | 2,142 (22.1) | |  |
| Regional hospital | 462 (29.0) | 2,318 (33.6) | 1,264 (31.1) | 3,317 (34.2) | |  |
| County hospital | 588 (37.0) | 2,349 (34.0) | 1,235 (30.4) | 3,045 (31.4) | |  |
| Private clinic | 155 (9.7) | 476 (6.9) | 478 (11.8) | 1,191 (12.3) | |  |
| **Annual volumes** |  |  |  |  | |  |
| Mean (SD) | 20 (15) | 30 (27) | 22 (15) | 31 (33) | |  |
| Median (Q1, Q3) | 15 (9, 28) | 24 (11, 38)) | 18 (10, 33) | 23 (13, 39) | |  |
| Min/Max | 1 /58 | 1 /141 | 1 /62 | 1 /205 | |  |
| **Annual volumes, n (%)** | | | | | |  |
| 0-10 | 464 (29.2) | 1,554 (22.5) | 1,084 (26.7) | 1,855 (19.1) | |  |
| 11-30 | 755 (47.5) | 2,974 (43.1) | 1,648 (40.5) | 4,255 (43.9) | |  |
| >30 | 372 (23.4) | 2,376 (34.4) | 1,334 (32.8) | 3,585 (37.0) | |  |
| **Values are n (%) unless otherwise stated.** | | | | | |  |
